# Delayed Antibody Response in the Acute Phase of Infection Is Associated with a Lower Mental Component of Quality of Life in Survivors of Severe and Critical COVID-19

**DOI:** 10.3390/jcm13071938

**Published:** 2024-03-27

**Authors:** Mohammad Mahmud Otman Dababseh, Peter Sabaka, Oľga Duraníková, Simona Horváthová, Peter Valkovič, Igor Straka, Anna Nagyová, Vladimír Boža, Marián Kravec, Ján Jurenka, Alena Koščálová, Peter Mihalov, Eliška Marešová, Matej Bendžala, Alice Kušnírová, Igor Stankovič

**Affiliations:** 1 Department of Infectology and Geographical Medicine, Faculty of Medicine, Comenius University in Bratislava, 833 05 Bratislava, Slovakia; m.dababseh@gmail.com (M.M.O.D.); nagyova324@uniba.sk (A.N.); jan.jurenka@gmail.com (J.J.); mihalovpeter@gmail.com (P.M.); elikrajcovicova@gmail.com (E.M.); mbendzala@gmail.com (M.B.);; 2 2nd Department of Neurology, Faculty of Medicine, Comenius University in Bratislava, 833 05 Bratislava, Slovakia; olga.duranikova@gmail.com (O.D.); simonah09@gmail.com (S.H.); peter.valkovic@fmed.uniba.sk (P.V.); straka0105@gmail.com (I.S.);; 3 Institute of Normal and Pathological Physiology, Slovak Academy of Sciences, 814 38 Bratislava, Slovakia; 4 Department of Applied Informatics, Faculty of Mathematics, Physics and Informatics, Comenius University in Bratislava, 842 48 Bratislava, Slovakiakravec18@uniba.sk (M.K.); 5 Department of Infectology, Slovak Medical University, 833 05 Bratislava, Slovakia; alena.koscalova@kr.unb.sk

**Keywords:** anti-spike SARS-CoV-2 antibodies, delayed antibody response, COVID-19, quality of life, mental health

## Abstract

**Background:** The long-term sequelae of coronavirus disease 2019 (COVID-19) significantly affects quality of life (QoL) in disease survivors. Delayed development of the adaptive immune response is associated with more severe disease and a worse prognosis in COVID-19. The effects of delayed immune response on COVID-19 sequelae and QoL are unknown. **Methods:** We conducted a prospective study to assess the relationship between the delayed antibody response in the acute phase of infection in naïve unvaccinated patients suffering from severe or critical COVID-19 and their QoL 12 months after hospital discharge. The 12-item Short Form Survey (SF-12) questionnaire was used for assessment of QoL. The SF-12 evaluates both mental and physical components of QoL, incorporating a mental component score (MCS-12) and a physical component score (PCS-12). A delayed antibody response was defined as testing negative for anti-spike SARS-CoV-2 antibodies at the time of hospital admission. **Results:** The study included 274 patients (154 men and 120 women). Of the enrolled patients, 144 had a delayed immune response. These patients had a significantly lower MCS-12 (*p* = 0.002), but PCS-12 (*p* = 0.397) was not significantly different at the 12-month follow-up compared to patients with positive anti-spike SARS-CoV-2 antibodies. The MCS-12 at the time of follow-up was negatively associated with delayed antibody response irrespective of possible confounders (*p* = 0.006; B = 3.609; ηp2 = 0.035; 95% CI = 1.069–6.150). An MSC-12 below 50 points at the time of follow-up was positively associated with delayed antibody response (*p* = 0.001; B = 1.092; OR = 2.979; 95% CI = 1.554–5.711). **Conclusions:** This study confirmed that, in patients with severe and critical COVID-19, a negative result for anti-spike SARS-CoV-2 antibodies at the time of hospital admission is associated with a lower mental component of QoL in unvaccinated patients naïve to COVID-19 one year after hospital discharge.

## 1. Introduction

The acute clinical presentation of coronavirus disease 2019 (COVID-19) ranges from asymptomatic or mild upper respiratory infection to life-threatening interstitial pneumonia leading to acute respiratory failure requiring oxygen therapy or even mechanical ventilation [1]. In most cases, COVID-19 resolves spontaneously without significant sequelae. However, up to 10% of COVID-19 survivors experience persistent symptoms beyond 12 weeks of infection [2]. Persistence of symptoms in COVID-19 survivors is known as long-COVID-19, or post-acute sequelae of SARS-CoV-2 infection (PASC). Although the symptoms of PASC are usually mild, up to 5% of patients suffer from symptoms that are severe enough to require medical care [3]. COVID-19 survivors also have a higher risk of developing severe cardiovascular, pulmonary, neurological, and psychiatric diseases, which may further impair their physical and mental health [4]. Persistence of symptoms affects the quality of life (QoL) of COVID-19 survivors, and the extent of the impact of COVID-19 sequelae on QoL correlates with the number and severity of persistent symptoms [5,6,7,8,9,10]. This affects both the physical and mental health of COVID-19 survivors. There is evidence that the impact on the mental components of QoL is even greater than on its physical components [8,9,10,11]. Specific humoral immunity plays a crucial role in recovery from COVID-19. The delayed dynamics of the antibody response to SARS-CoV-2 have been identified as a predictor of poor outcomes, possibly impairing the ability to fully recover from the disease. An association between delayed antibody response and disease severity and the extent of organ damage has been proposed [12,13]. The impact of delayed antibody response on QoL in COVID-19 survivors is yet to be determined.

## 2. Materials and Methods

### 2.1. Design

This is a prospective observational study which aims to identify the effects of a delayed anti-spike SARS-CoV-2 antibody response in unvaccinated and previously COVID-19-naïve patients hospitalised with COVID-19 on their quality of life assessed 12 months (±14 days) after discharge from the hospital. A delayed anti-spike SARS-CoV-2 antibody response was defined as testing negative for anti-spike SARS-CoV-2 IgG antibodies at the time of hospital admission.

### 2.2. Patients

All patients admitted to the Department of Infectology and Geographical Medicine, University Hospital in Bratislava, Slovakia and to the Faculty of Medicine, Comenius University in Bratislava, Slovakia during the period from 1 April 2020 to 30 December 2021 were eligible for consideration of enrolment. The inclusion criteria were: COVID-19 with acute SARS-CoV-2 infection confirmed by polymerase chain reaction for SARS-CoV-2 RNA on nasopharyngeal swab on hospital admission; a severe or critical course of COVID-19 as the reason for hospitalisation (defined by the National Institutes of Health (NIH) guidelines [14]); age < 75 years; and the ability to answer the questions in the QoL questionnaire. The exclusion criteria were a self-reported history of COVID-19 and vaccination against SARS-CoV-2 infection. At the time of admission, clinical and anthropometric data were obtained. Their medical history was obtained by questionnaire. Subsequently, venous blood was drawn from the cubital vein to measure blood count and the concentrations of creatinine, C-reactive protein (CRP), D-dimer, and interleukin-6 (IL-6). Venous blood was also collected into EDTA to assess the presence of anti-spike SARS-CoV-2 IgM and IgG antibodies. The patients were then followed up for 12 months ± 14 days after hospital discharge by phone call to assess their quality of life. The 12-item Short Form Survey (SF-12) questionnaire was used for quality of life assessment at the follow-up. The questionnaires were evaluated according to the provider manual [15]. The SF-12 is a well-established general health questionnaire designed as the shorter form of the standard 36-item Short Form Survey (SF-36) questionnaire. The SF-12 uses 12 questions drawn from each of the eight dimensions of the SF-36. It is designed to have similar performance to the SF-36 while being less time consuming to complete and being better suited to follow-up via telephone. The SF-12 evaluates both mental and physical components of QoL as a mental component score (MCS-12) and a physical component score (PCS-12). This questionnaire is regarded to be an accurate and valuable tool for the evaluation of QoL and its results are consistent with the SF-36 [16,17,18]. All patients suffering from severe or critical COVID-19 were treated by 6 mg of dexamethasone daily. In patients requiring high-flow nasal oxygen or mechanical ventilation, 4 mg of oral baricitinib was added to the corticosteroid therapy, per institutional guidelines.

### 2.3. Biochemical Analysis

Immunoturbidimetry (Cobas Integra 400, Roche Diagnostics, Rotkreuz, Switzerland) was used to measure CRP and D-dimer concentrations in serum. Serum creatinine concentrations were measured using spectrophotometry (Cobas Integra 400, Roche Diagnostics, Rotkreuz, Switzerland). Concentrations of IL-6 were measured using an immunoassay (Elecsys, Roche Diagnostics, Rotkreuz, Switzerland). The presence of anti-spike SARS-CoV-2 IgG and IgM antibodies in serum was assessed using a lateral flow assay (COVID-19 IgG/IgM Rapid Test, Zhejiang Orient Gene Biotech Co., Ltd., Huzhou, China). A total of 5 μL of serum was added to the test slide, followed by 80 μL of the buffer. The results of tests were read after 15–20 min according to the manufacturer’s recommendations. The same laboratory technician read all of the tests to minimise the risk of bias. Tests in which the control line didn’t appear after 15 min were regarded as invalid.

### 2.4. Statistical Analysis

Initially, a Kolmogorov–Smirnov test was used to assess the normality of distributions of the quantitative variables. None of the variables were normally distributed. Quantitative variables were therefore expressed as medians and the 25th and 75th percentiles. The medians of the quantitative variables were compared using a Mann–Whitney U test. The distributions of the categorical variables among the study groups were assessed using a chi-square test. The associations between the mental and physical QoL component scores with other variables were assessed using a multivariate linear regression. The variables associated with the presence of anti-spike SARS-CoV-2 antibodies at baseline with *p*-values below 0.01 in univariate analysis were included in the multivariate model. These variables were duration of symptoms, prior admission, neutrophil-to-lymphocyte ratio, CRP, fasting glucose concentration, and glomerular filtration. We assessed the associations between the mental and physical component scores of QoL below 50 points and other variables using binary logistic regression. Probability values (*p*-values) less than 0.05 were considered statistically significant. Odds ratios (OR) and 95% confidence intervals (95% CI) were used to quantify the strength of the associations between covariates and dependent variables in multivariate models. SPSS version 20 (IBM Corp., Armonk, NY, USA) was used for statistical analysis. The figures were created using GraphPad Prism version 10 (Dotmatics Corp., Boston, MA, USA).

### 2.5. Ethics

The study was carried out in accordance with the Declaration of Helsinki. All participants were fully informed regarding the purpose of the study and potential risks of participation. Written informed consent was obtained from all participants before enrolment (Protocol S1). All of the data used in the analysis were fully anonymised.

## 3. Results

**Figure 1:**
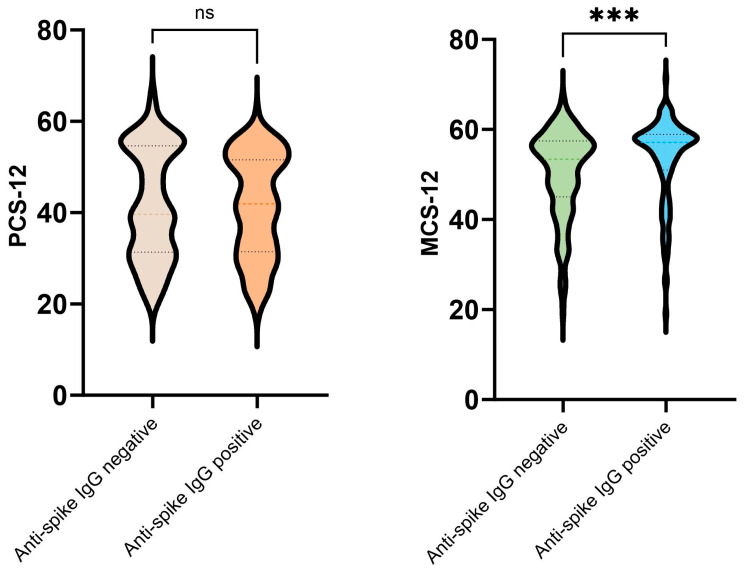
The mental component score-12 and physical component score-12 of the SF-12 quality of life questionnaire at the time of follow-up. IgG—immunoglobulin G, MCS-12—mental component score-12, ns—not significant, PCS-12—physical component score-12. ***—*p* value = 0.0001.

The study included 274 patients overall (154 men and 120 women) who met the inclusion criteria and were eligible for QoL assessment via telephone. At the time of hospital admission, 552 patients were screened for study enrolment. Of those patients, 106 died (78 patients died during their stay in hospital and 28 died during the 1 year follow-up period), and 172 patients were unable or unwilling to answer the questions in the QoL questionnaire or were unable to be reached by telephone. Of the enrolled patients, 144 had negative and 130 had positive anti-spike SARS-CoV-2 IgG antibodies at the time of hospital admission. The baseline characteristics of the patients are provided in Table 1. The patients with negative anti-spike SARS-CoV-2 IgG antibodies at the time of admission had significantly lower CRP concentrations and neutrophil to lymphocyte (N/L) ratios. They also experienced a shorter duration of symptoms prior to admission. Patients with negative anti-spike SARS-CoV-2 IgG antibodies were younger and had a lower fasting glucose at admission; however, the difference was not statistically significant. There was no difference in BMI or Charlson comorbidity index between the groups. The proportions of patients of male gender; those admitted to ICU; and those with critical disease were also not significantly different between groups (Table 1). Patients with negative anti-spike SARS-CoV-2 IgG antibodies at the time of admission had a significantly lower MCS-12 at the time of follow-up, but there was no significant difference in PCS-12 (Table 2, Figure 1). MCS-12 at the time of follow-up was positively associated with the presence of anti-spike SARS-CoV-2 IgG antibodies at the time of admission irrespective of age, GFR, CRP, N/L ratio, fasting glucose, or time from disease onset to hospital admission (Table 3). Having an MSC-12 of less than 50 points at the time of follow-up was positively associated with having negative anti-spike SARS-CoV-2 IgG at the time of hospital admission, irrespective of age, GFR, CRP, N/L ratio, fasting glucose, or time from disease onset to hospital admission (Table 4).

## 4. Discussion

### 4.1. Main Findings

In our cohort, the absence of detectable anti-spike SARS-CoV-2 IgG antibody production at the time of hospital admission was associated with a lower mental component QoL score in unvaccinated, SARS-CoV-2 infection naïve COVID-19 patients one year after hospital discharge. This association was irrespective of age, comorbidity, the time from disease onset to antibody assessment, and other possible confounders. To our knowledge, this study is the first to describe this association.

### 4.2. Pathophysiological Background

The timing and strength of the immune response in COVID-19 has a significant impact on the disease course and outcomes. There is evidence that a delayed humoral immune response is associated with more severe disease, higher in-hospital mortality, and higher mortality after hospital discharge [12,13]. Despite the known association between delayed immune response and poor outcomes, there has been a lack of evidence linking a delayed immune response with mental components of QoL in COVID-19 survivors. There is a positive correlation between disease severity and the risk of development of PASC [19]. Persistence of symptoms of PASC subsequently impairs the QoL of COVID-19 survivors [5,6,7,8,9,10]. If PASC develops, the mental component of QoL is affected to the same or even to a greater extent than the physical component [10,11]. Jacobs et al. [8] reported significantly poorer mental health in COVID-19 survivors with PASC. Todt et al. [6] also reported a decreased mental component of QoL and an increased prevalence of anxiety and depression in patients with PASC. However, it is unclear if the impairment of the mental component of QoL in patients with PASC correlates with disease severity. McFann et al. [9] reported greater limitations in social functioning in survivors of severe disease compared to those who had mild disease. They also observed more severe limitations due to emotional problems. They reported no effect on emotional wellbeing, however. Their study was limited by a small number of participants. Despite that, they reported an association between disease severity and all physical aspects of QoL. Rass et al. [10] also found no association between disease severity and the mental component of QoL. In our study, we found no associations between ICU admission or the presence of critical disease and the presence of anti-spike SARS-CoV-2 IgG antibodies at the time of hospital admission. We were also unable to confirm the association of delayed antibody response with impaired physical aspects of QoL. There is evidence that regulation (and deregulation) of the immune response to SARS-CoV-2 may affect the mental health of COVID-19 survivors. Deregulation of the immune response with exaggerated inflammation in the lung parenchyma plays a crucial role in the pathogenesis of severe COVID-19. It is also responsible for a wide variety of pathological immunological phenomena associated with acute and post-acute COVID-19 and is associated with high levels of proinflammatory cytokines and inflammatory markers like CRP [20,21]. Dispinseri et al. [22] concluded that a delayed antibody response in COVID-19 is a major trait of immune response deregulation in severe COVID-19. In our study, we found lower concentrations of proinflammatory markers such as CRP and the N/L ratio in patients with negative anti-SARS-CoV IgG antibodies at the time of hospital admission. Dispinseri et al. [22] also concluded that delayed production of antibodies in COVID-19 is associated with lower CRP and N/L ratio at the time of acute disease. Jurenka et al. [13] reported lower CRP and Il-6 concentrations and lower N/L ratios in patients with negative anti-spike SASR-CoV-2 antibodies at the time of hospital admission; despite this, these patients had a significantly worse long-term prognosis. In our study, there was no association of CRP concertation or N/L ratio with the mental component of QoL. Thus, we are unable to conclude that hyperinflammation at the time of hospital admission is associated with mental health impairment in survivors. However, we assessed CRP and N/L ratio only at the time of hospital admission and didn’t assess the dynamics of inflammatory markers throughout the hospitalisation. Hyperinflammatory response in the acute phase of infection is likely not directly responsible for mental health impairment in patients with delayed immune responses. However, the deregulation of the immune response in COVID-19 is complex and goes beyond simple hyperinflammation [23]. Encephalopathy and encephalitis associated with COVID-19 and PASC are often mediated by the production of neural autoantibodies. These conditions may subsequently lead to various neuropsychiatric symptoms [24]. Franke et al. [25] found neuronal autoantibodies in more than 50% of COVID-19 survivors with persistent cognitive impairment. Deregulation of the immune response to SARS-CoV-2 infection is considered to be one of the main drivers of autoimmunity in COVID-19 [26]. Other, more direct pathological mechanisms leading to neurologic and psychiatric sequelae of COVID-19 have been proposed [27]. SARS-CoV-2 is able to invade human endothelial cells. Direct invasion to the microvascular endothelium by COVID-19 weakens the blood-brain barrier and may exacerbate the local inflammatory response [28]. The virus also infects microglia, leading to an inflammatory response at the level of the brain parenchyma [29]. There is evidence that the SARS-CoV-2 virus has the ability to directly invade neurons. However, it is currently believed that this mechanism likely plays a secondary role in the development of neurological symptoms of PASC and that the indirect effects of infection on neuroinflammation as described above hold much greater significance [30]. Virus-neutralising anti-spike antibodies block the interaction of virions with angiotensin converting enzyme 2 (ACE2) receptors [31]. This not only prevents cell invasion, limiting the extent of infection, but also counteracts the viral effect on ACE2 activity. An infection-mediated decrease in ACE2 activity stimulates the inflammatory response and likely plays a crucial role in deregulation of the local immune response [32]. Rapid development of the anti-spike antibody response may therefore limit the extent of neural invasion and also diminish its proinflammatory effects in the brain tissue.

On the other hand, antibody production and dynamics of antibody response might be influenced by pre-existing mental conditions. There is evidence that the presence of depressive syndrome negatively affects the antibody response to a second dose of anti-SARS-CoV-2 vaccine in healthcare workers [33]. A large meta-analysis revealed negative impacts of psychological stress on the antibody response to influenza vaccination [34].

Even a short course of corticosteroid therapy may induce significant mood and cognitive changes. However, these effects are reversible in most patients [35]. It is possible that corticosteroid therapy used to treat COVID-19 and its side effects might contribute to the QoL impairment in some of the survivors of COVID-19. But based on the evidence about limited duration of corticosteroid side effects in the majority of treated patients, we suggest that these effects did not significantly affect our results.

Our results further support the mounting body of evidence that the dynamics and character of the immune response contribute to the impairment of mental health and the mental components of QoL in COVID-19 survivors. It appears that the rapid development of the immune response in the acute phase of COVID-19 is crucial not just for survival, but also to limit the neuropsychiatric sequelae subsequently affecting QoL.

### 4.3. Practical Implications

The dynamics of the immune response to infection by the SARS-CoV-2 omicron variant are affected by previous vaccination. Vaccinated individuals develop antibodies against SARS-CoV-2 sooner than unvaccinated individuals, and recipients of a 3rd booster dose have faster and more vigorous antibody responses than recipients of the original two-dose vaccination course. In vaccinated individuals, the dynamics of the antibody response negatively correlates with increasing time since the last dose of the COVID-19 vaccine [36]. In light of these findings, our results further support the role of the COVID-19 vaccination program in the prevention of PASC and QoL decline associated with COVID-19 sequelae.

### 4.4. Limitations

This study has several limitations. First, we used only qualitative methods for antibody detection. We therefore could not assess the possible correlation of QoL with antibody titres. We used point-of-care lateral flow serological tests because they are a faster, less expensive, and less elaborate method compared to enzyme-linked immunoassays (ELISA). We were unable to use more elaborate methods such as ELISA because of limited human and financial resources during the COVID-19 pandemic. The point-of-care test used in our study has a sensitivity of >98% and a specificity of 100% relative to the ELISA with a detection limit of 0.4 units per ml and is considered to be an accurate alternative for quantitative detection of anti-spike antibodies [37]. Second, the study design (recruitment of all eligible consecutive patients) prevented exact matching of the cohorts for age, gender, and the Charlson comorbidity index. However, the cohorts did not differ in these variables; we therefore suggest that this limitation did not affect our results. We used multivariate analyses to diminish the effects of possible confounders. Third, the time from disease onset to admission was shorter in patients with negative anti-spike SARS-CoV-2 antibodies, which may affect our assessment of the dynamics of immune response. However, there were no associations between the time from disease onset to hospital admission (and blood sampling) and the mental aspects of QoL in the multivariate model. Fourth, we didn’t assess the socioeconomic status of participants. There is evidence that socioeconomic deprivation is positively associated with the perception of symptoms of long COVID-19 [38]. Therefore, we are unable to exclude the potential bias created by socioeconomic inequalities between our participants. Fifth, according to evidence, the prevalence of PASC is not distributed homogenously across the population and mostly affects middle-aged patients [39]. The limited number of participants in our study prevented us from subgrouping the patients according to age. There may be different effect sizes of the antibody dynamics on the mental aspects of PASC; however, the relatively low number of subjects in our study prevented us from their determination.

## 5. Conclusions

Negative anti-spike SARS-CoV-2 IgG antibodies at the time of hospital admission were associated with worse mental QoL scores in survivors of severe and critical COVID-19 when assessed 12 months after hospital discharge. This suggests that a delayed anti-spike antibody response promotes the development of neuropsychiatric sequelae in COVID-19 survivors. However, further studies are needed to clarify the role of the delayed dynamics of antibody response in the pathogenesis of mental impairment in the post-acute sequelae of SARS-CoV-2 infection.

## Figures and Tables

**Table 1:** Baseline characteristic of patients.

Continuous Variables		Anti-Spike SARS-CoV-2 IgG Negative (*n* = 144)	Anti-Spike SARS-CoV-2 IgG Positive (*n* = 130)	*p*-Value
Age	years	54 (45, 65)	59 (51, 67)	0.094
CCI		2 (2, 2)	2 (1, 2)	0.572
BMI	kg/m^2^	29 (26, 34)	29 (26, 33)	0.918
Duration of symptoms	days	7 (6, 9)	10 (7, 11)	0.0001
CRP	mg/L	58 (23, 112)	118 (58, 165)	0.0001
GFR	mL/min	113 (85, 140)	102 (80, 136)	0.022
N/L ratio		4 (3, 7)	6 (4, 9)	0.0001
Fasting glucose	mmol/L	6 (6, 8)	7 (6, 8)	0.087
Length of hospital stay	days	10 (7, 13)	8 (6, 13)	0.254
Categorical variables		anti-spikeARS-CoV-2 IgG positive, *n*/*n*-total (%)	anti-spikeARS-CoV-2 IgG negative, *n* (%)	*p*-value (chi-square)
Gender	Male	77 (53.47%)	77 (59.23%)	0.338
	Female	67 (46.53%)	53 (40.77%)	
ICU admission	Yes	25 (17.36%)	15 (11.54%)	0.230
	No	119 (82.64%)	115 (88.46%)	
Critical disease	Yes	23 (15.97%)	17 (13.08%)	0.278
	No	121 (84.03%)	113 (86.62%)	

BMI—body mass index, CCI—Charlson Comorbidity Index, CRP—C-reactive protein, GFR—glomerular filtration rate, ICU—intensive care unit, n—number of subjects, N/L ratio—neutrophil to lymphocyte ratio, *p*—probability.

**Table 2:** The mental component score-12 and physical component score-12 of the SF-12 quality of life questionnaire at the time of follow-up.

Variable	Anti-Spikepike SARS-CoV-2 IgG Negative	Anti-Spikepike SARS-CoV-2 IgG Positive	*p*-Value
PCS-12	39.62 (31.36, 54.61)	41.92 (31.42, 51.56)	0.397
MCS-12	53.37 (45.01, 57.09)	57.10 (50.96, 58.87)	0.0003
Categorical variables	anti-spike SARS-CoV-2 IgG positive, *n*/*n*-total (%)	anti-spike SARS-CoV-2 IgG negative, *n* (%)	*p*-value (chi-square)
PCS-12 below 50	94 (65.27%)	85 (65.38%)	1.000
MCS-12 below 50	58 (40.28%)	29 (22.31%)	0.002

MCS-12—mental component score-12, *p*-value—probability, PCS-12—physical component score-12.

**Table 3:** The multiple linear logistic regression of association of mental component score-12 of the SF-12 quality of life questionnaire with other variables.

Variable	*p*	B	η*p*2	95% CI
Anti-spike SARS-CoV-2 IgG positive	0.006	3.609	0.035	1.069–6.150
Age	0.076	−0.101	0.014	−0.213–0.011
CRP	0.480	−0.006	0.002	−0.023–0.011
GFR	0.689	−0.007	0.001	−0.042–0.028
N/L ratio	0.256	0.125	0.006	−0.091–0.340
Fasting glucose	0.813	−0.048	0.0001	−0.451–0.335
Duration of symptoms	0.750	0.046	0.000	−0.241–0.334

B—Unstandardised regression weight, CI—Confidence interval, CRP—C-reactive protein, GFR—Glomerular filtration rate, *p*—Probability, N/L—Neutrophil to Lymphocyte ratio.

**Table 4:** The binary logistic regression of association of a mental component score-12 of less than 50 points with other variables.

Variable	*p*	B	OR	95% CI
Anti-spike SARS-CoV-2 IgG positive	0.001	1.092	2.979	1.554–5.711
age	0.414	0.012	1.012	0.984–1.041
CRP	0.920	0.000	1.000	0.996–1.005
GFR	0.381	−0.004	0.996	0.987–1.005
N/L ratio	0.878	−0.004	0.996	0.944–1.145
Fasting glucose	0.813	−0.048	1.037	0.939–1.145
Duration of symptoms	0.627	−0.21	0.979	0.900–1.065

B—Unstandardised regression weight, CI—Confidence interval, CRP—C-reactive protein, GFR—Glomerular filtration rate, *p*—Probability, N/L—Neutrophil to Lymphocyte ratio, OR—Odds Ratio.

## Data Availability

The data used in this study are available on request from the corresponding author on reasonable request.

## Supplementary Materials

The following supporting information can be downloaded at: https://www.mdpi.com/article/10.3390/jcm13071938/s1, Protocol S1: Ethics Committee statements (protocols: KR 022020, 30 March 2020 and KR 182023, 27 June 2023).
