# Supplementary material for: Delayed Antibody Response in the Acute Phase of Infection Is Associated with a Lower Mental Component of Quality of Life in Survivors of Severe and Critical COVID-19
Source: J Clin Med. 2024 Mar 27;13(7):1938. doi: 10.3390/jcm13071938 (PMC11012816; doi:10.3390/jcm13071938)
Supplement: Supplementary file 1 [file jcm-13-01938-s001.zip › jcm-2891893-supplementary.pdf]

Stanovisko etickej komisie ku klinickej štúdii

Statement of the Ethics Committee to a clinical study

**Názov a adresa etickej komisie**<br>*Name and address of the Ethics Committee*	Etická komisia UN Bratislava, Nemocnica akad. L. Dérera<br>Limbová 5<br>833 05 Bratislava<br>Slovenská republika
**Meno a adresa skúšajúceho**<br>*Name and address of the investigator*	doc. MUDr. Peter Sabaka, PhD.<br>Klinika infektológie a geografickej medicíny<br>Limbová 5<br>Bratislava 83305<br>Slovenská republika
**Názov štúdie**<br>**COVID- 19 UNB**	
**Sponzor štúdie/ zástupca sponzora v SR**<br>*Sponsor of the trial / sponsor's representative in SR*	
**Kód štúdie** / *Study code*	

Etická komisia posúdila žiadosť o štúdiu na základe priložených dokumentov:

Ethics Committee reviewed the application for the clinical trial according to following documents:

**Priložené dokumenty** / *Submitted documents:*	**Číslo, verzia, dátum vydania** / *Number, version, date:*
**Protokol štúdie**<br>*Study protocol*	4 strany
**Súhrn protokolu**/*Protocol synopsis*	
**Dodatok k protokolu**<br>*Protocol amendment*	
**Informácia o lieku pre skúšajúceho**<br>*Clinical investigator's brochure*	
**Informácia pre pacienta a informovaný súhlas**<br>*Patient information and informed consent*	1 strana
**Poistenie štúdie**<br>*Insurance*	
**Záznamník údajov o pacientovi**<br>*Case report form*	
**Iné dokumenty** / *Other documents:*<br>Žiadosť o súhlas s realizáciou výskumu<br>Životopis hlavného skúšajúceho - doc. MUDr. Sabaka<br>Súhlas vedenia pracoviska KIGM (doc.MUDr. Stankovič)<br>Základný formulár pre neanimálne experimenty	 dodané<br>dodané<br>dodané

Pripomienky / *Comments:*

MUDr. Bilíková- Ak budú pacienti s COVID 19 hospitalizovaní na iných oddeleniach,
je potrebný súhlas od vedenia dotknutých klinik.

súhlasím

Dátum 21. 4. 2020 **podpis / pečiatka** [signature]

Date *Signature / stamp*

Predseda etickej komisie

Chairman of the Ethics Committee

Etická komisia zohľadňuje požiadavky Správnej klinickej praxe (ICH-GCP), Štátneho ústavu pre kontrolu liečiv (ŠÚKL) a platnej legislatívy pre klinické skúšanie v SR.

The Ethics Committee complies with principles of ICH-GCP and applicable regulatory requirements.

Klinické skúšanie sa môže vykonať len so súhlasom Štátneho ústavu pre kontrolu liečiv a príslušného zdravotníckeho zariadenia.

The trial may be realized only with approval of the State Institute of Drug Control and of particular healthcare provider.

Zoznam prítomných členov etickej komisie je prílohou tohto dokumentu. Hlasovali len členovia etickej komisie nezávislí od sponzora štúdie. Skúšajúci (pokiaľ bol prítomný) sa hlasovania nezúčastnil.

A list of present members of the Ethics Committee is attached. Only those Ethics Committee members who are independent of the sponsor of the trial have voted. The investigator (if he/she was present) has not participated in the vote of the Ethics Committee.

Stanovisko etickej komisie k biomedicínskemu výskumu

Statement of the Ethics Committee to a biomedical research

č. rozhodnutia: 18 /2023

**Názov a adresa etickej komisie**<br>*Name and address of the Ethics Committee*	Etická komisia UN Bratislava, Nemocnica akad. L. Dérera<br>Limbová 5<br>833 05 Bratislava<br>Slovenská republika
**Meno a adresa skúšajúceho**<br>*Name and address of the investigator*	doc.MUDr. Peter Sabaka. PhD.<br>Klinika infektológie a geografickej medicíny LF UK a UNB<br>Nemocnica akad.L.Dérera<br>Limbová 5<br>831 01 Bratislava<br>Slovenská republika
**Názov štúdie**<br><br>**COVID-19 - Imunity and quality of life in convalescent patients**	
**Sponzor štúdie/ zástupca sponzora v SR**<br>*Sponsor of the trial / sponsor's representative in SR*	Lekárska fakulta UK v Bratislave<br><br>Ústav klinického a translačného výskumu<br>Biomedicínske centrum SAV
**Kód štúdie** / *Study code*	

Etická komisia posúdila žiadosť o výskum na základe priložených dokumentov:

Ethics Committee reviewed the application for the research according to following documents:

**Priložené dokumenty** / *Submitted documents:*	**Číslo, verzia, dátum vydania** / *Number, version, date:*	
**Protokol štúdie**<br>*Study protocol*	3 strany	dodané
**Súhrn protokolu**/*Protocol synopsis*		
**Dodatok k protokolu**<br>*Protocol amendment*		
**Informácia o lieku pre skúšajúceho**<br>*Clinical investigator's brochure*		
**Informácia pre pacienta a informovaný súhlas**<br>*Patient information and informed consent*	1 strana	dodané
**Poistenie štúdie**<br>*Insurance*		
**Záznamník údajov o pacientovi**<br>*Case report form*		
**Iné dokumenty** / *Other documents:*		
Žiadosť o stanovisko Etickej komisie		dodané
Životopis hlavného skúšajúceho- doc.Sabaka		dodané
Základný formulár pre neanimálne experimenty	2 strany	dodané
Súhlas vedenia KIGM LFUK a UNB		dodané

Etická komisia ~~SÚHLASÍ~~ / SÚHLASÍ S PRIPOMIENKAMI (uvedené nižšie) / ~~NESÚHLASÍ (dôvody uvedené nižšie)~~ s vykonaním uvedeného biomedicínskeho výskumu .

*Ethics Committee ~~**AGREES**~~ / **AGREES WITH COMMENTS** (stated below) / ~~**DISAGREES** (reasons stated below)~~ with submitted biomedical research to be realized.*

Pripomienky / *Comments:*

Doplnenie informovaného súhlasu:

dobrovoľná účasť v projekte, právo subjektu odvolať svoj súhlas s účasťou v projekte resp. kedykoľvek z projektu odstúpiť

spôsob nakladania s dátami účastníkov- dôvernosť dát, súlad s GDPR

Dodať dotazník alebo súbor otázok pre telefonické opytovanie účastníkov o kvalite života

Dokumentáciu žiadame doplniť do 31.7.2023.

UNIVERZITNÁ NEMOCNICA BRATISLAVA
Nemocnica akad. L. Derera
Limbová 5, 833 05 Bratislava
ETICKÁ KOMISIA -1-

Dátum 27. 6. 2023
Date

podpis / pečiatka
Signature / stamp

Predseda etickej komisie
Chairman of the Ethics Committee

Odôvodnenie: Predložená dokumentácia spĺňa požiadavky Etickej komisie a je v súlade s platnou legislatívou
Rationale: Enclosed documents comply with the requirements of the Ethics committee and in accordance with law.

Etická komisia zohľadňuje požiadavky Správnej klinickej praxe (ICH-GCP), Štátneho ústavu pre kontrolu liečiv (ŠÚKL) a platnej legislatívy pre klinické skúšanie v SR.
The Ethics Committee complies with principles of ICH-GCP and applicable regulatory requirements.

Klinické skúšanie sa môže vykonať len so súhlasom Štátneho ústavu pre kontrolu liečiv a príslušného zdravotníckeho zariadenia.
The trial may be realized only with approval of the State Institute of Drug Control and of particular healthcare provider.

Zoznam prítomných členov etickej komisie je prílohou tohto dokumentu. Hlasovali len členovia etickej komisie nezávislí od sponzora štúdie. Skúšajúci (pokiaľ bol prítomný) sa hlasovania nezúčastnil.
A list of present members of the Ethics Committee is attached. Only those Ethics Committee members who are independent of the sponsor of the trial have voted. The investigator (if he/she was present) has not participated in the vote of the Ethics Committee.

Zoznam členov etickej komisie

A list of members of the Ethics Committee

Dátum zasadania/Meeting date: 27.6.2023

Názov a adresa etickej komisie:

Name and address of the Ethics Committee: Etická komisia UN Bratislava
Nemocnica akad.L.Dérera
Limbová 5
833 05 Bratislava
Slovenská republika

**Meno**/*Name*	**Titul**/*Title*	**Povolanie**/*Occupation*	**Pracovisko**/*Working place*	**Prítomný**/*Present*
Bilíková Katarína	MUDr. Mgr.	lekár predseda EK	Odd.klin.farmakológie UNB Ružinov	[illegible]
Tibenský Igor	MUDr . PhD.,MBA	námestník pre zdr.starostlivosť	UNB Kramáre	
Slimáková Ľubica	PharmDr. MPH.	farmaceut	Lekáreň UNB Kramáre	[illegible]
Paštrnáková Viera	PhDr. MPH.	námestník OŠ	UNB Kramáre	[illegible]
Rimovská Mária	JUDr.	právnik	Právne oddelenie UN Bratislava	[illegible]
Demeš Martin	Doc.,MUDr., PhD.,MPH	internista	I. Interná klinika UNB Kramáre	[illegible]
Šusterová Adriána	Mgr.	farmaceut tajomník EK	Lekáreň UNB Kramáre	[illegible]
Petríčková Helena	JUDr.	právnik	t.č. v dôchodku	[illegible]

UNIVERZITNÁ NEMOCNICA
BRATISLAVA
Nemocnica akad. L. Dérera
Limbova 5, 833 05 Bratislava
ETICKÁ KOMISIA
